# Immunosuppression Efficacy via Nonlinear Modeling and Probabilistic Analysis of Human Leukocyte Antigen Match and Dose Effects on Allograft Rejection Risk

**DOI:** 10.1002/iid3.70302

**Published:** 2025-11-26

**Authors:** Samrajya Raj Acharya, Aayush Man Regmi, Sushil Ghimire, Ram Prasad Ghimire

**Affiliations:** ^1^ Department of Mathematics, School of Science Kathmandu University Dhulikhel Nepal; ^2^ Department of Mathematics VIT University Vellore India

## Abstract

**Background:**

Human Leukocyte Antigen (HLA) compatibility is a key driver of kidney allograft outcomes, yet how matching interacts with immunosuppressant dosing remains unclear. This study evaluates whether low‐resolution HLA match fractions affect mean dosing and quantified the joint impact of match quality and dose on rejection risk.

**Methods:**

In 519 transplants grouped by HLA match (0–1.0 in 7 strata), full data completeness and variance homogeneity is confirmed, followed by the usage of one‐way ANOVA to compare mean doses. Ridge‐penalized logistic regression models was fitted at each match level to predict rejection probability across low‐ and high‐dose regimens, deriving absolute and relative risk reductions.

**Results:**

ANOVA showed no significant dosing differences by match (*F*_6,512_ = 0.93, *p* = 0.47). Logistic models revealed that Absolute Risk Reduction rose from ~0.75% at zero match to ~1.25% at perfect match, while mid‐range match groups (0.2–0.6) yielded the highest total prevented rejections under high‐dose therapy.

**Conclusion:**

Uniform baseline dosing eliminates bias, allowing clear attribution of benefit to immunologic compatibility. These findings endorse a risk‐tailored immunosuppression approach intensifying doses where match is low and potentially reducing them in highly compatible transplants and identify match ranges that maximize rejection prevention.

## 1 Introduction

Kidney transplantation offers a life‐saving option for patients with end‐stage renal disease but host graft immunologic incompatibilities continue to drive graft loss and patient morbidity. Human Leukocyte Antigen (HLA) compatibility plays a vital role in kidney transplantation outcomes, influencing both graft survival and rejection risk and mortality with HLA disparities exerting the greatest impact on long‐term outcomes. While prior research has highlighted the benefits of improved HLA matching, the precise relationship between donor–recipient compatibility, immunosuppressant dosing, and rejection risk requires further quantitative exploration. In this study, a data set consisting of posttransplant cases, stratified by discrete HLA match fractions is analyzed to investigate how varying levels of compatibility affect immunosuppressant requirements and the likelihood of rejection.

Following transplantation, patients are placed on a standardized immunosuppressive regimen typically a combination of two or three drug classes to keep rejection risk within acceptable bounds. The central challenge is to quantify how incremental changes in drug exposure translate into changes in rejection probability while balancing the risks of under‐ and over‐immunosuppression.

Recent studies in renal transplantation have increasingly used mathematical and statistical models to better understand and identify how genetic differences between donors and recipients, especially mismatches in HLA, presence of Donor‐Specific Antibodies (DSA) and Panel Reactive Antibodies (PRA), and the levels of immunosuppressive drugs given posttransplantation, affect the risk of transplant rejection. These models include basic methods like logistic regression and Cox proportional hazards models, as well as more advanced techniques such as penalized regression and machine learning algorithms.

Williams et al. [1] performed a cohort study of first adult deceased‐donor kidney transplants, using a Cox proportional hazards model to assess how HLA mismatches (A, B, DR) influence graft survival finding that each additional HLA mismatch huddled a statistically significant increase in the threat of transplant failure, showcasing the critical importance of donor–recipient HLA compatibility for long‐term graft outcomes. Studies concerning HLA mismatch data with details of immunosuppressive drug exposure have embraced a systems‐level modeling approach. Gao et al. [2] used logistic model, that included HLA‐B eplet mismatch, delayed graft function, and tacrolimus levels after 12 months, achieved a predictive accuracy of ~0.73, showing a rational performance. Wong et al. [3], in a Southeast Asian population‐based data, stratified patients into risk groups based on their eplet mismatch levels and adjusted for different immunosuppressive regimens in their Cox model. Even after accounting for drug type: tacrolimus and cyclosporine, the genetic mismatch categories remained significant predictors of survival without rejection.

Leeaphorn et al. [4] analyzed data from over 93,000 kidney transplants and found that mismatches in HLA‐DQ significantly raised the risk of acute rejection and lowered graft survival, suggesting that closer HLA‐DQ matching should be prioritized during transplant allocation to improve outcomes. Magga et al. [5] noted that, although Cox proportional‐hazards models are widely used to identify risk factors for kidney graft failure, they can overfit when many predictors compete with limited sample sizes; consequently, they highlight the need for automated, regularized variable selection methods in transplant survival research.

Lee et al. [6] integrated both genetic and pharmacological data to predict the development of de novo donor‐specific antibody (dnDSA), which are early warning signs of rejection. Using Cox regression, they found that high HLA Class‐II eplet mismatch combined with low tacrolimus exposure significantly raised the hazard of developing these antibodies. This signifies the value of incorporating diverse patient data to enhance accuracy in calculating risk. Some machine learning models and analyses have also advanced, for instance, Xia et al. [7] developed a de‐biased LASSO approach for stratified Cox models, designed to manage complex, high‐dimensional transplant data sets applied to national kidney transplant data, successfully identifying important risk factors for graft failure across varying donor and recipient age groups. Davis et al. [8] investigated the role of HLA‐DR/DQ molecular mismatch in predicting the development of dnDSAs during the initial year after transplantation. The researchers found that adequate tacrolimus exposure can adjust the impact of HLA Class‐II molecular mismatch, suggesting that maintaining therapeutic tacrolimus levels may mitigate the risk associated with HLA incompatibility.

Nemati et al. [9] applied a range of machine learning algorithms including Coxnet, a penalized Cox model, random survival forests, and gradient boosting to over 100,000 kidney transplant cases, converting categorical HLA types into numerical features using methods like eplet counts and target encoding that allowed the models to learn complex patterns in the data. While the improvement in predictive accuracy, measured by the concordance index, was modest, it confirmed the value of incorporating genetic mismatch data into survival prediction models.

Mankowski et al. [10] highlighted the ongoing restriction between promoting equity in deceased‐donor kidney allocation and improving transplant outcomes through HLA matching by emphasizing emerging evidence that eplet mismatches are a finer‐grained measure than traditional antigen mismatches can better predict immunological risk, motivating the need to integrate eplet‐level data into organ allocation policies. Li et al. [11] highlighted that mismatched HLA eplets and antigens play a crucial role in the development of de novo DSAs and antibody‐mediated rejection after renal transplantation. It underscores the requirement to better understand which specific eplet mismatches influence immunological outcomes to improve graft survival in transplanted patients.

These regression models typically include predictors such as the count of mismatched HLA types and drug concentrations, and they output either odds, in logistic models or hazard rates in Cox models of experiencing rejection. The consistent finding across multiple studies is that both high HLA mismatch and subtherapeutic immunosuppression independently contribute to poor transplant outcomes. Massie et al. [12] pointed out that although kidney transplantation generally provides better survival outcomes than dialysis, the emergence of the COVID‐19 pandemic introduced significant new risks for immunosuppressed patients. Such circumstances created uncertainty in clinical decision‐making, especially since earlier studies did not incorporate models that reflected the dynamic nature of the pandemic. For this, researchers explored stochastic simulation and machine learning to explore when kidney transplantation continues to offer a benefit and when it might pose greater risks during uncertain and rapidly changing conditions like those seen during pandemic. Alves et al. [13] emphasized that traditional HLA matching does not fully capture immunological compatibility and introduces HLA eplet mismatch load as a more precise metric for predicting graft outcomes. It highlights prior findings that link higher eplet mismatches with increased risk of DSA and graft rejection, justifying the study's focus on evaluating this association in living donor kidney transplantation.

Aforementioned studies demonstrate how modeling techniques can be adapted to clinical data to provide meaningful insights in immunity and immunosuppression. While prior studies have examined the impact of HLA mismatches or immunosuppressant dosing separately, few have jointly quantified how their interaction modifies rejection risk. Lim et al. [14] found that HLA mismatches significantly increased the risk of rejection, graft failure, and victimization independent of the initial immunosuppressive dosage, underscoring the lack of integrated models assessing compatibility and medication. When predictors are numerous and correlated with each other, logistic regression models probabilities, Cox models time‐to‐event outcomes, and penalized models are useful. Across all methods, a clear result emerges: both genetic mismatches and insufficient drug therapy are key, independent predictors of rejection. Combining both types of data improves the ability to predict transplant outcomes. This interdisciplinary research area illustrates how mathematical modeling can play a critical role in improving medical decision‐making and patient care.

In this study, a dataset of transplant cases stratified into low‐resolution HLA (A, B, DRB1) match‐fraction groups to assess whether match level influences mean immunosuppressant dosage and how compatibility and dose jointly impact rejection probability is analyzed from a transplant center in Nepal. Although recent studies emphasize eplet‐level mismatches as a more granular predictor of immunologic risk, such high‐resolution data are not routinely available in many settings, including Nepal. This study therefore evaluates rejection risk using low‐resolution HLA match fractions, providing a practical framework that can inform clinical decision‐making where only standard typing is feasible, while still drawing insights consistent with the broader literature on eplet mismatches. A multistage statistical approach of hypothesis testing after confirming data completeness and variance homogeneity despite modest departures from normality is employed, that found no significant dosage differences by match fraction via one‐way analysis of variance (ANOVA). Constrained, ridge‐penalized logistic regression is applied across each match level to model rejection risk as a function of dose and to derive absolute and relative risk reductions (RRR) between lower and upper dosing bounds. The ridge‐penalized logistic regression is chosen as compared to traditional models since it stabilizes coefficient estimates in modestly sized subgroups and reduces the risk of overfitting. The results quantify how incremental improvements in HLA matching translate into clinically meaningful reductions in rejection risk, informing both individualized dosing strategies and organ‐allocation policies.

## 2 Methodology

The data set represents transplant cases recorded over the decade period ending in beginning of 2024, yielding 519 complete observations for analysis. Immunosuppressant dose was quantified as the daily tacrolimus dose administered to each patient measured in milligrams (mg), ranging from 20 to 300 mg right after transplant. Immunosuppressant dosing was standardized and monitored immediately posttransplant, ensuring that variations in drug exposure did not confound the analysis. This comprises observations with low‐resolution HLA match fractions that have been seen as the match with the respective donor, which are A, B, and DRB1, followed by their antigen types I and II. The match fractions go as 0/6, 1/6, 2/6, 3/6, 4/6, 5/6, and 6/6 matches categorized for ease of analysis as 0, 0.17, 0.33, 0.5, 0.67, 0.83, and 1.0, respectively, for this study. While the mathematical model has been validated, the unavailability of long‐term posttransplant data on biopsy reports or clinician‐documented suspicion limits practical validation. For this reason, the manuscript focuses on mathematical modeling that examines associations between HLA match fractions and initial immunosuppression dosing patterns. Other medical constraints like DSA and PRA are treated as constant.

**Table 1:** Test for data completeness.

Variables	Observations	Missing	Valid (%)
Immunosuppressant	519	0	100
Match Levels	519	0	100

The transplant rejection risk is accesed as a modeled probability of rejection, calculated separately for each group of HLA matching. This analysis relied on a logistic regression model that connected the patient's baseline immunologic compatibility with their initial daily tacrolimus dose. To keep the predicted probabilities realistic and avoid overfitting, the models incorporated constrained optimization along with ridge (*L2*) regularization. With the help of these models, key risk measures are calculated, along with its confidence intervals or error bands. This approach enabled to quantify how incremental changes in HLA matching or immunosuppressant dosing might influence rejection risk, providing a basics for individualized dosing strategies and inform decisions on organ allocation. A one‐way ANOVA was employed to compare mean immunosuppressant dosages across these groups. To ensure the validity of the ANOVA, preliminary checks for missing values, normality, and homogeneity of variances were conducted. This section presents the methodology, results, and interpretations of these statistical tests in detail. Prior to statistical analysis, it is essential to verify the completeness of the data set to avoid biases or errors in subsequent tests which is shown in Table 1.

No missing values were identified in either column, confirming a complete data set of 519 observations suitable for analysis. The Shapiro–Wilk test assesses whether the immunosuppressant dosages within each match fraction group follow a normal distribution, a key assumption for parametric tests like ANOVA.

Null Hypothesis (H_0_): The immunosuppressant dosages within each match fraction group are normally distributed.

Alternative Hypothesis (H_1_): The immunosuppressant dosages within each match fraction group are not normally distributed.

The Shapiro–Wilk test was applied individually to the dosage data within each of the seven match fraction groups, with significance assessed at α = 0.05 as shown in Table 2.

**Table 2:** Shapiro–Wilk test for normality test.

Match level	Shapiro–Wilk *p*
0	0.001
0.17	0.001
0.33	0.001
0.5	0.001
0.67	0.001
0.83	0.001
1	0.001

In Table 2, for all match fraction groups, the *p* values are < 0.05, leading to the rejection of the null hypothesis. This indicates that the immunosuppressant dosages within each group deviate significantly from a normal distribution. However, ANOVA is known to be robust to violations of normality, particularly with large sample sizes. Thus, while this assumption is not met, the overall impact on the ANOVA results is likely minimal due to the sample size and group balance. Levene's test for homogeneity is further conducted that evaluates whether the variances of immunosuppressant dosages are equal across the match fraction groups, a critical assumption for the validity of ANOVA as is presented in Table 3.

**Table 3:** Levene's test for homogeneity.

Test	df_1_	df_2_	*F*	*p*
Levene's	6	512	0.71	0.649

Null Hypothesis (H_0_): The variances of immunosuppressant dosages are equal across all match fraction groups.

Alternative Hypothesis (H_1_): At least one match fraction group has a variance that differs from the others.

The *p* value of 0.6494 exceeds the significance threshold of α = 0.05, leading to a failure to reject the null hypothesis. This result suggests that the variances of immunosuppressant dosages are statistically equal across all match fraction groups. The satisfaction of this assumption supports the appropriateness of proceeding with the one‐way ANOVA. The one‐way ANOVA tests whether there are significant differences in the mean immunosuppressant dosages across the seven match fraction groups as presented in Table 4.

**Table 4:** One‐way ANOVA test for variance.

Source	SS	df	MS	*F*	*p*
Between groups	12,391.47	6	2065.25	0.933	0.4708
Within groups	1,133,377	512	2213.27	—	—
Total	1,145,768.47	518	—	—	—

Null Hypothesis (H_0_): The mean immunosuppressant dosages are equal across all match fraction groups.

Alternative Hypothesis (H_1_): At least one match fraction group has a mean immunosuppressant dosage that differs from the others.

The ANOVA yielded an *F*‐statistic of 0.932969 and a *p* value of 0.47075. Since the *p* is > 0.05, we fail to reject the null hypothesis. This indicates that there is no statistically significant difference in the mean immunosuppressant dosages across the match fraction groups. The lack of significance suggests that the match fraction does not appear to influence the dosage administered.

By first confirming with ANOVA that the average immunosuppressive dose did not differ across HLA matches (*p* > 0.05), this establishes that dose allocation was uniform and therefore cannot confound the findings. This validation step allows to proceed with the constrained, regularized logistic risk modeling with confidence that any observed variations in rejection risk can be reflected in shifts in the intercept which is baseline risk and slope which is dose sensitivity are driven by true immunologic differences in HLA compatibility rather than by systematic differences in how patients were dosed. In other words, because dosing was equivalent across match strata, the logistic model's estimates cleanly disentangle the independent effects of match and dose on rejection probability.

### 2.1 Logistic Risk Modeling

Transplant rejection risk is often modeled as a binary outcome (reject/no reject) with predictors including immunosuppressive dose and donor–recipient compatibility. In kidney transplantation, higher HLA match is known to reduce rejection risk. The probability of rejection is modeled using logistic regression, where the logistic function (sigmoid) maps a linear predictor into (0,1). Specifically, for dose *x* and parameters $(b_{0}$, $b_{1}$), the equation is:

$$p\left(x\right)=\frac{1}{1+\text{exp}\left(-\left(b_{0}+b_{1}x\right)\right)}.$$

To ensure realistic risk predictions, constrained optimization on ($b_{0}$, $b_{1})$ with bounds such that the predicted risk never falls below 0.001 at both low‐ and high‐dose extremes is used. This avoids implausibly low probabilities at very high doses. The parameters are optimized separately for each discrete HLA match level from 0 to 1. Also, ridge (*L2*) regularization is included in the fitting objective to penalize large coefficients. In practice, the negative log‐likelihood plus a penalty term *λ* (*b*_0_^2^ + *b*_1_^2^) is minimized. The ridge penalty hyperparameter (*λ*) was chosen via grid search with cross‐validation to optimize model fit while controlling coefficient magnitude and preventing overfitting. The *λ* yielding the best cross‐validated performance was selected for the final models. In other words, the cost function was of the form:

$$L\left(b\right)=-\sum_{i}\left[y_{i}\ln\left(p\left(x_{i}\right)\right)+\left(1-y_{i}\right)\ln\left(1-p\left(x_{i}\right)\right)\right]+\lambda \left(b_{0}^{2}+b_{1}^{2}\right).$$

This shrinks the magnitude of *b*_0_ and *b*_1_ and helps prevent overfitting. The penalty hyperparameter *λ* was chosen to balance fit quality and parameter regularity. For each HLA match fraction, we report the fitted logistic parameters $(b_{0}$, $b_{1}$) and derived risk metrics. Denote the fitted risk at the lower dose *x*_min_ by *p*_min_ = *p*(*x*_min_) and at the upper dose *p*_max_ = *p*(*x*_max_). The computations are:

Absolute Risk Reduction (ARR): *p*_min_ − *p*_max._

Relative Risk Reduction (RRR): (*p*_min_ − *p*_max_)/*p*_min._

RRR percentage reduction: (ARR/low‐dose risk) × 100%.

Expected Rejection Reduction (ERR): *N* (*p*_min_ − *p*_max_).

## 3 Results and Discussion

**Figure 1:**
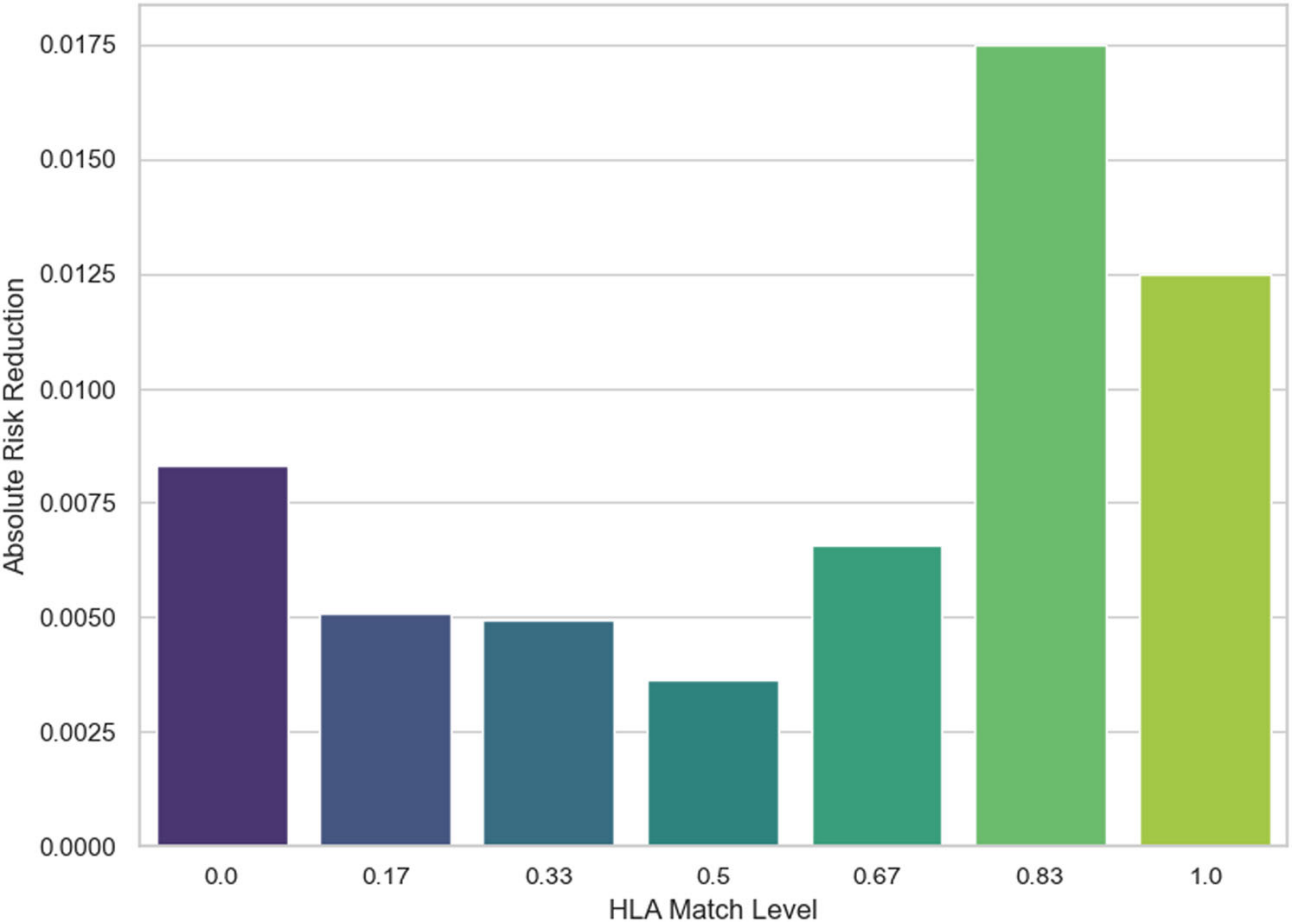
ARR versus HLA match level.

Figure 1 demonstrates that the calculation of ARR is the predicted probability of rejection on the low‐dose regimen minus that on the high‐dose regimen. ARR increases with HLA match, from effectively 0.75% benefit at 0 match to 1.25% reduction in risk at perfect match of 1, which is about 0.5% absolute risk difference at full match. A rising ARR implies that high‐dose immunosuppression confers more extra benefit. In contrast, when the match is poor, the model predicts that adding more drugs hardly lowers the already high baseline risk. For perfectly matched kidneys, high‐dose therapy might prevent ~0.4% of patients from rejecting. This suggests that for well‐matched recipients, the standard lower dose already keeps rejection risk very low, and the extra drugs yield only marginal benefit. These findings are consistent with the idea of risk‐tailored immunosuppression, where highly compatible transplants can be managed less aggressively.

**Figure 2:**
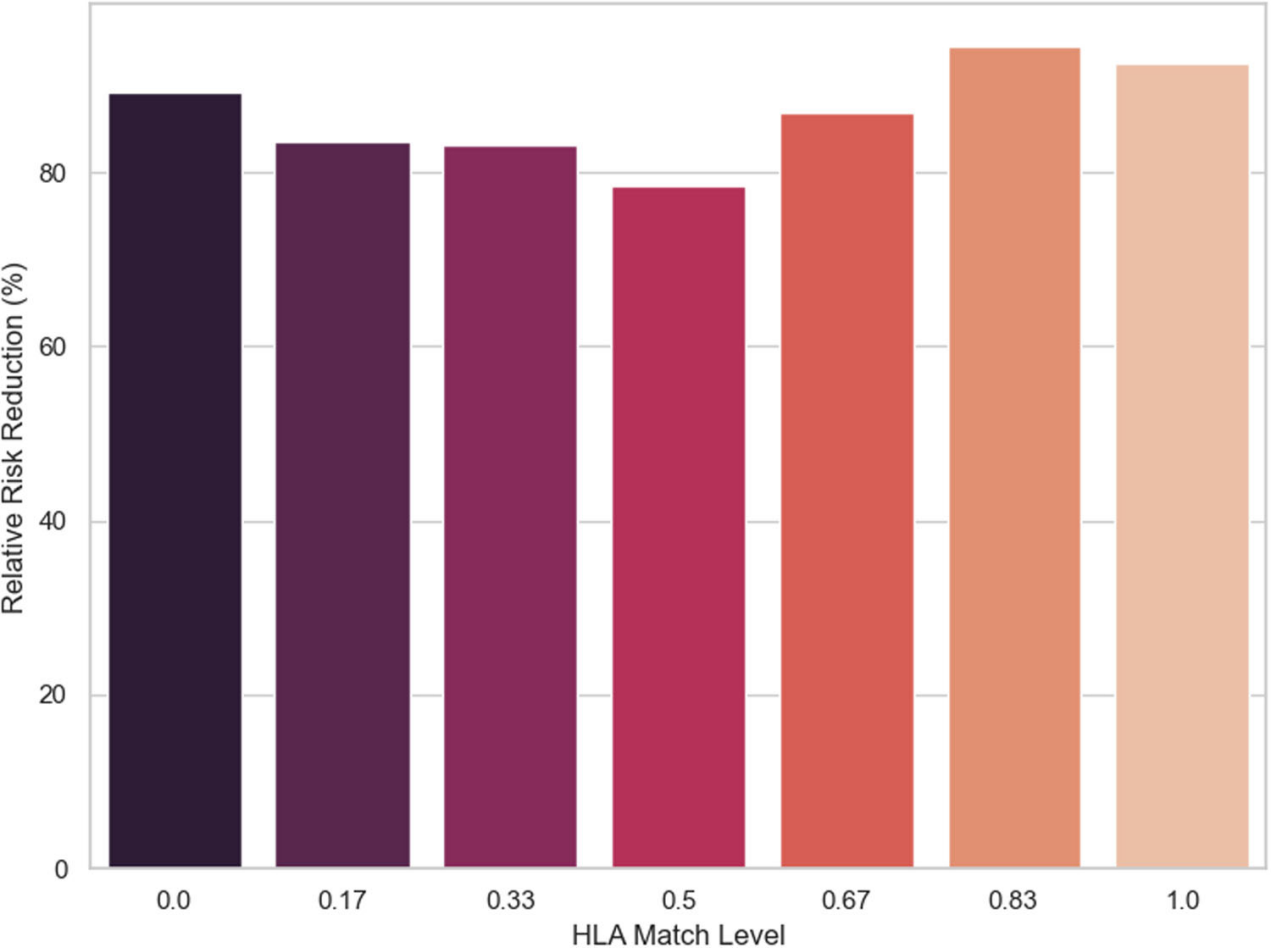
RRR versus HLA match level.

Figure 2 shows how RRR is calculated as the percent drop relative to baseline risk across match levels. RRR is at 85% at 0 matches, lowers slightly at mid‐matches, then rises to 95% at 0.83 match and 92% at perfect match. It expresses the fraction of the original risk that is eliminated by switching to a high dose. This signifies when match is high, the baseline risk is small, so eliminating it constitutes a large fraction, hence RRR is large. This suggests that for well‐matched patients, intensifying immunosuppression could dramatically improve relative safety. For poorly matched patients, the relative benefit is marginal, meaning that even a strong dose would not greatly change the high risk they face.

**Figure 3:**
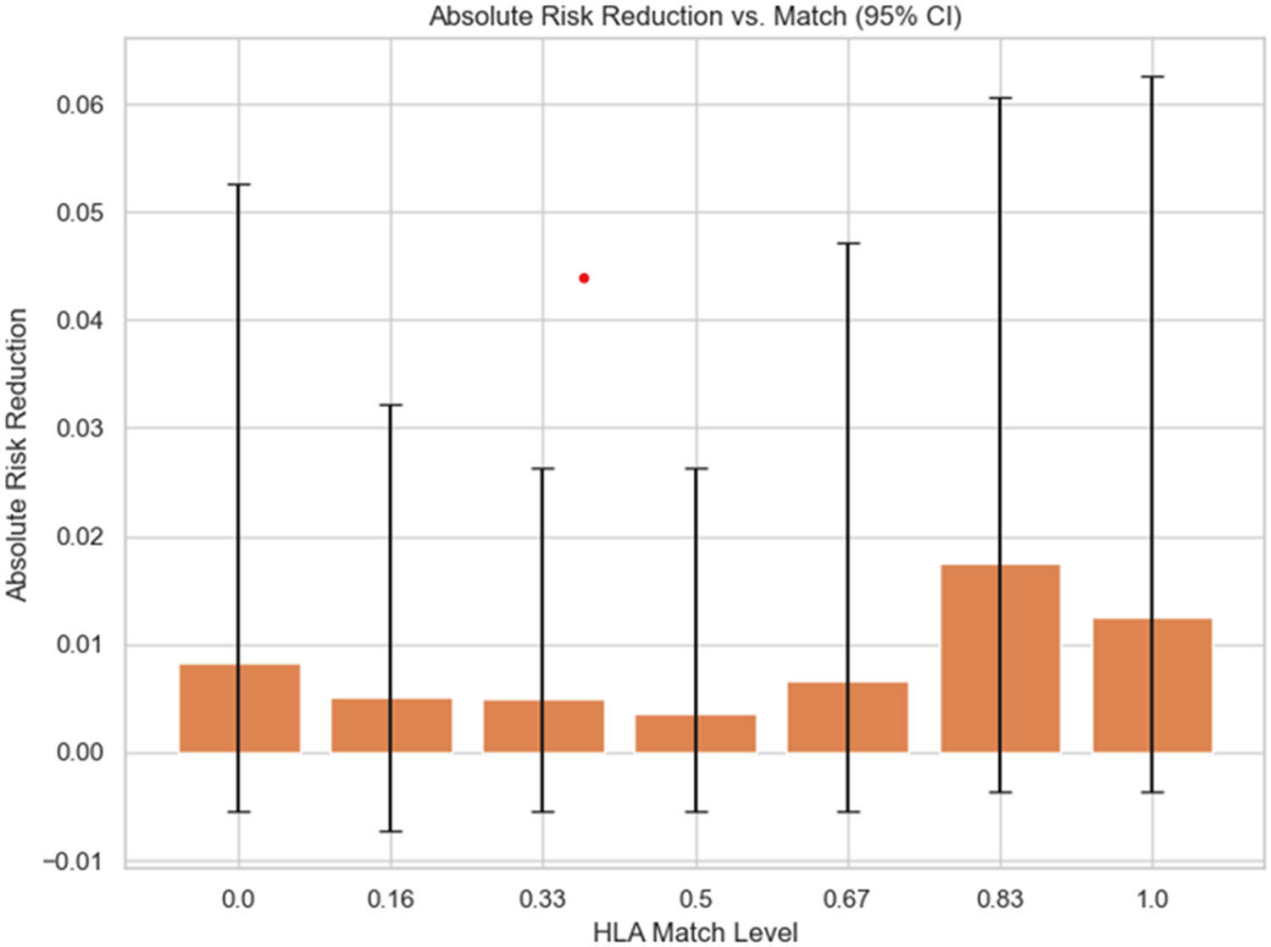
ARR with confidence interval (95%).

Figure 3 interprets ARR with 95% confidence intervals across HLA match levels where the mean ARR tends to rise as HLA compatibility improves, indicating greater potential benefit from higher‐dose immunosuppression in well‐matched transplants. However, the wide confidence intervals especially at very low and very high match levels show that the estimates are imprecise. In poorly matched kidneys, extra immunosuppression adds little consistent benefit, while in highly matched kidneys (≥ 0.83), the apparent advantage is larger but still uncertain. These results support the concept of tailoring immunosuppression to match quality, but also emphasize the need for caution given the variability in effect size.

**Figure 4:**
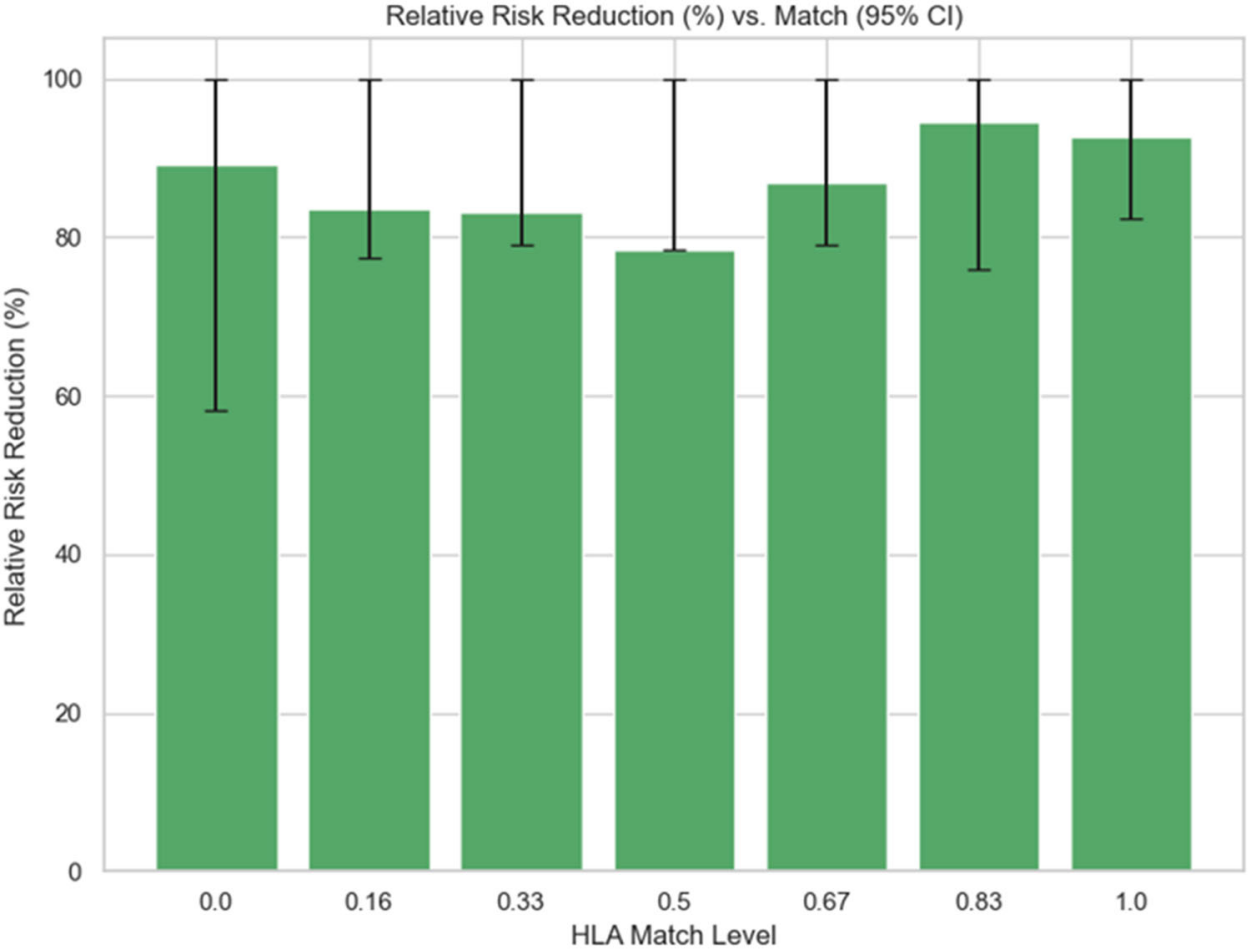
RRR with confidence interval (95%).

Figure 4 demonstrates the RRR with 95% confidence intervals across HLA match levels. The pattern shows that RRR is somewhat lower at mid‐match levels (0.33–0.5) but rises again as compatibility improves, reaching its highest values at 0.83 (5/6 match) and full match. The wide and overlapping confidence intervals indicate uncertainty, which suggests these differences cannot be considered statistically conclusive. This indicates that strong immunosuppression is most effective in patients with better HLA compatibility, where the baseline rejection risk is already lower. For poorly matched pairs, the potential gain from higher doses is smaller and more variable, implying limited practical benefit despite aggressive treatment.

**Figure 5:**
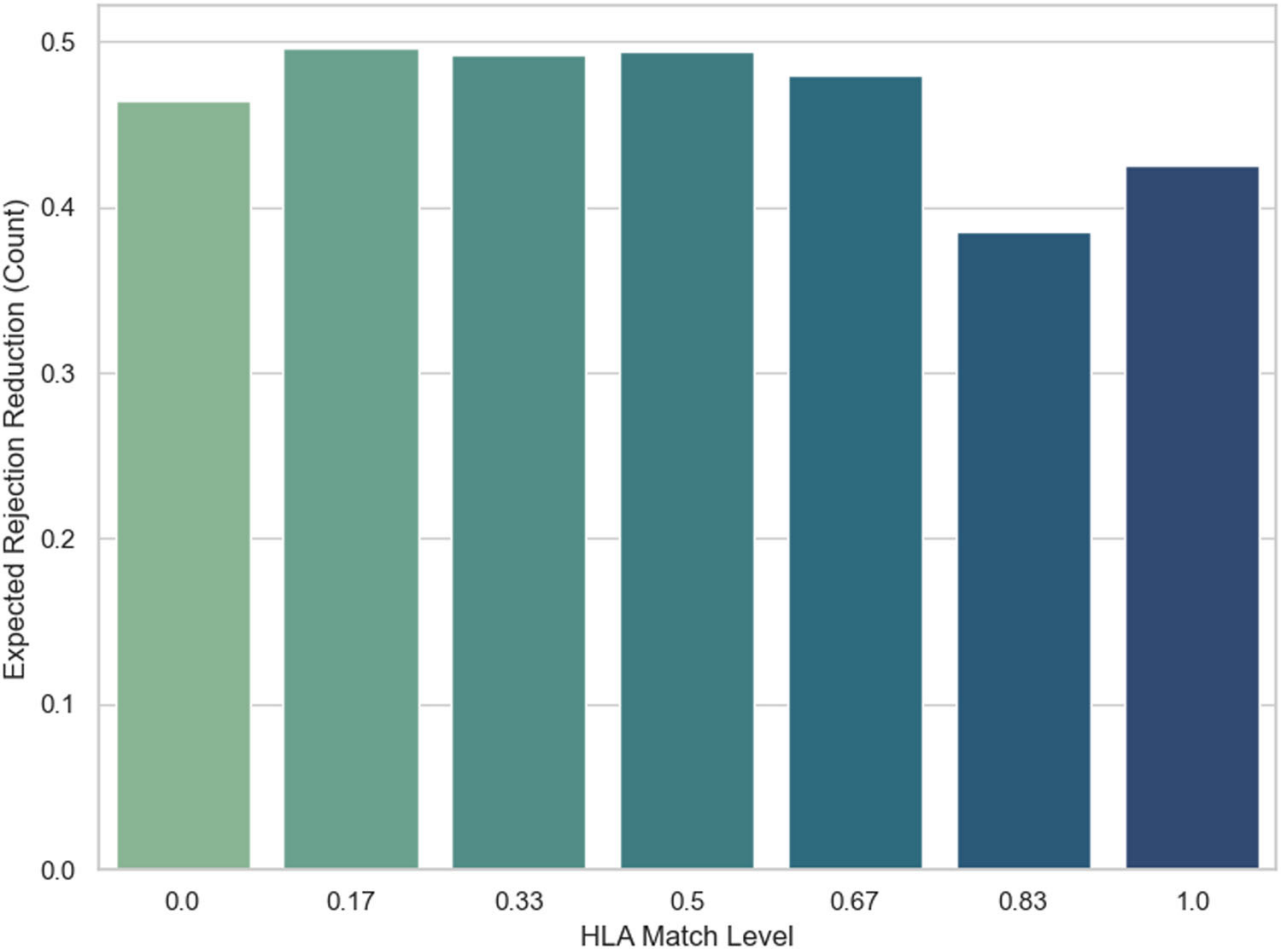
ERR versus HLA Match.

In Figure 5, ERR is calculated as the bars of prevented rejections achieved by using the high dose rather than the low dose. It essentially multiplies the ARR by the number of samples in each match level. This metric translates percentage reductions into actual event counts. It reflects that well‐matched patients, despite low baseline risk, still have enough risk of rejection. Even the best match only prevents about 40%–45% of kidney rejection but in contrast we can observe that lower matches have higher rejection reduction from 45% to 50%. This is occurring due to difference in numbers of sample in each of the match levels.

**Figure 6:**
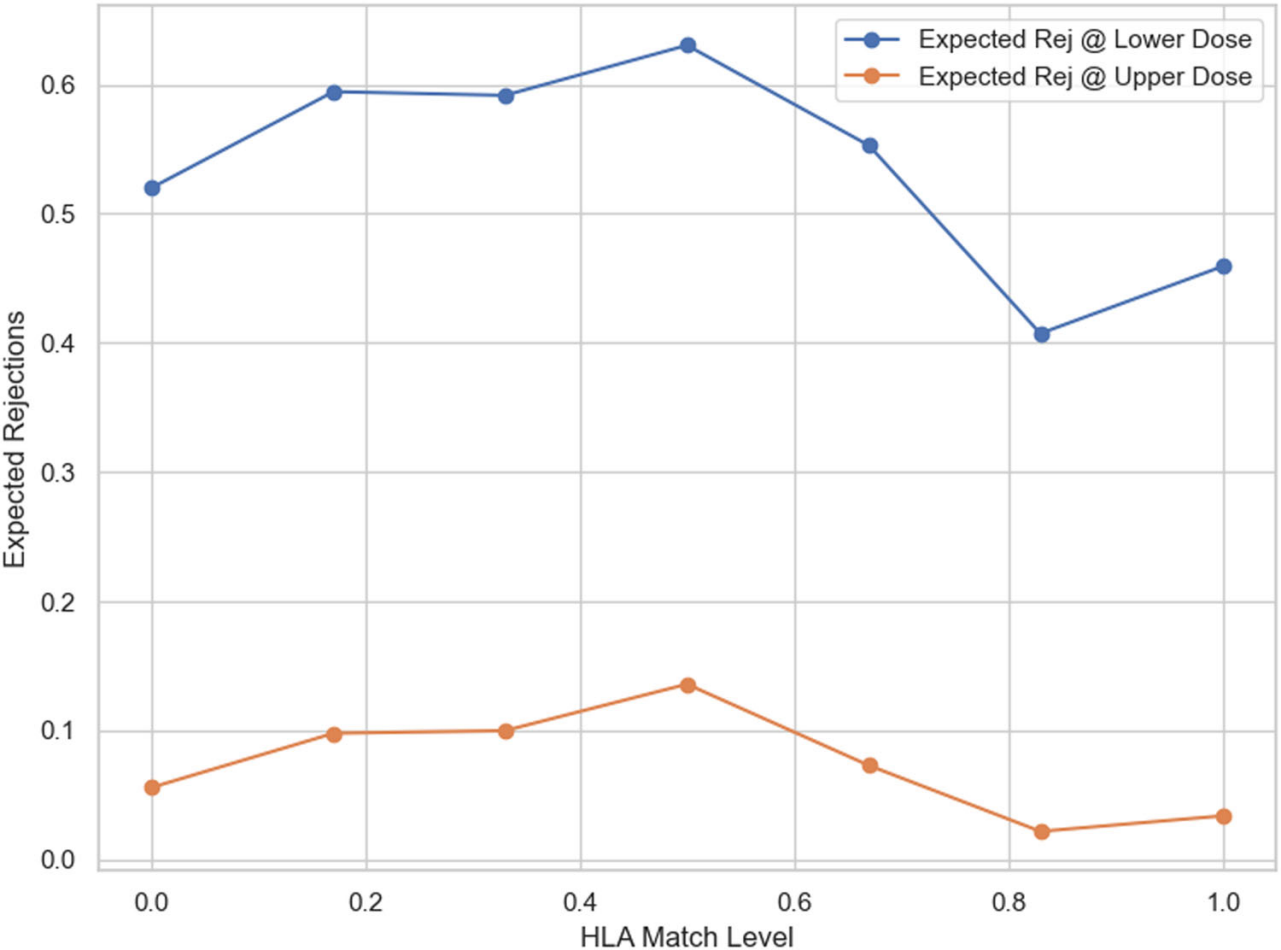
Expected rejections at lower versus upper dose.

Figure 6 illustrates the predicted number of rejection events at each match level for the lower and higher doses. At every level of HLA match, the expected rejections are higher under low dose than under high dose. The greatest absolute difference is at low match. The vertical gap between the curves at any given match is exactly the expected number of events prevented. As the match improves, total events drop sharply under both doses. This means that in a population with poorly matched kidneys, intensifying immunosuppression could prevent roughly half of the expected rejection. In contrast, in highly matched kidneys, there is a statistically low to no chance of rejection. In summary, high‐dose prescription lowers the expected rejection across all matches, but its practical impact is most pronounced when HLA mismatch is high.

**Figure 7:**
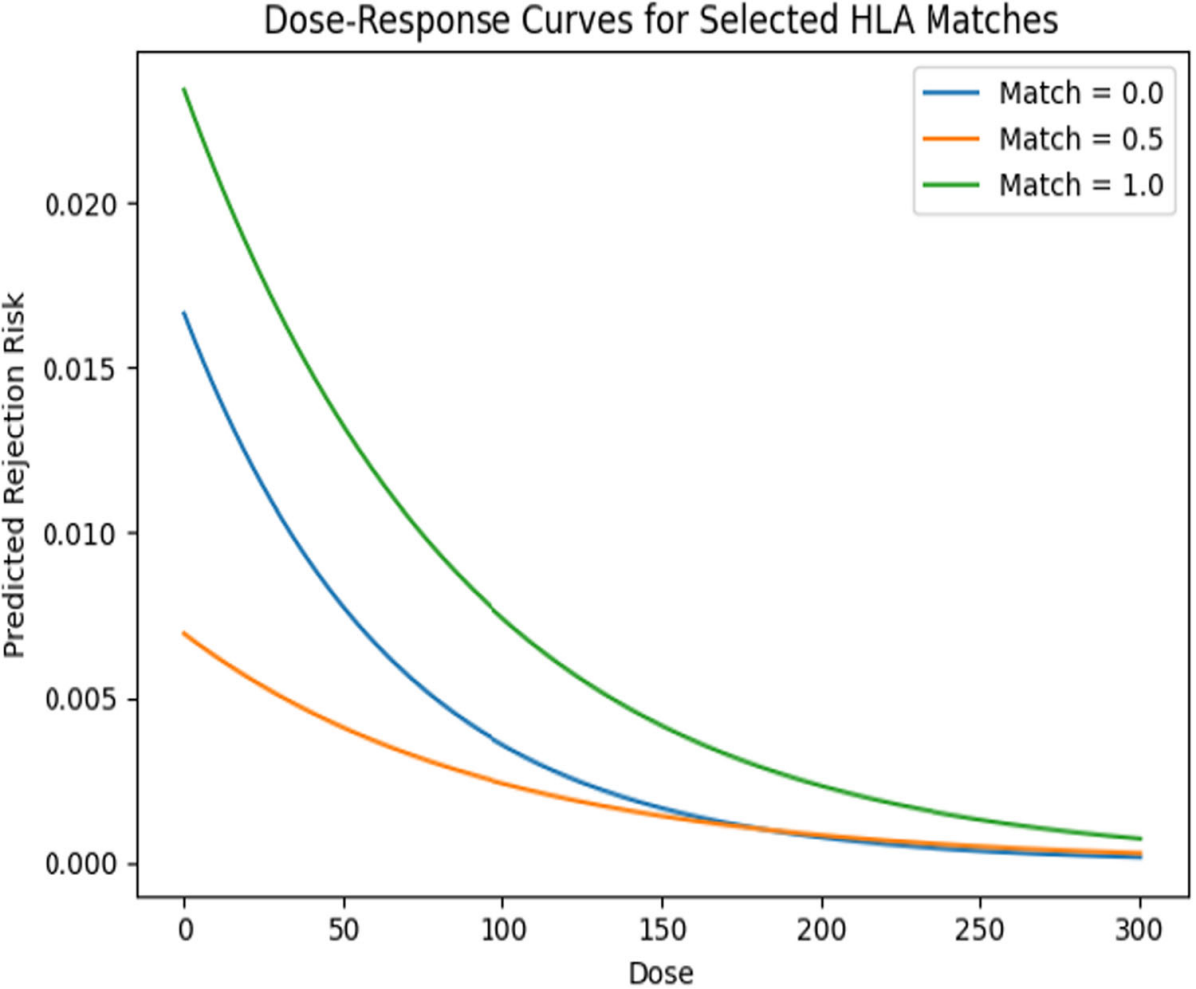
Logistic curves of risk rejection.

Figure 7 shows continuous logistic curves of rejection risk versus dose for match: 0.0, 0.5, and 1.0. Poor match of 0 starts at 0.017 risk at dose 0, declines steeply to 0.0003 at dose 300; 0.5 match starts lower 0.007 declines moderately. Whereas, perfect match of 1.0 starts highest 0.023, declines more slowly. This illustrates the full sigmoid response, showing that dose–response curves differ by match compatibility. Poor matches get big absolute drops at low‐dose increments, then flatten out. Perfect matches start at higher initial estimates due to sample size constraints, but still drop slower. This helps visualize where two observed doses sit on each curve and choose dosing strategies that land in the steepest part of the response for each match group.

**Figure 8:**
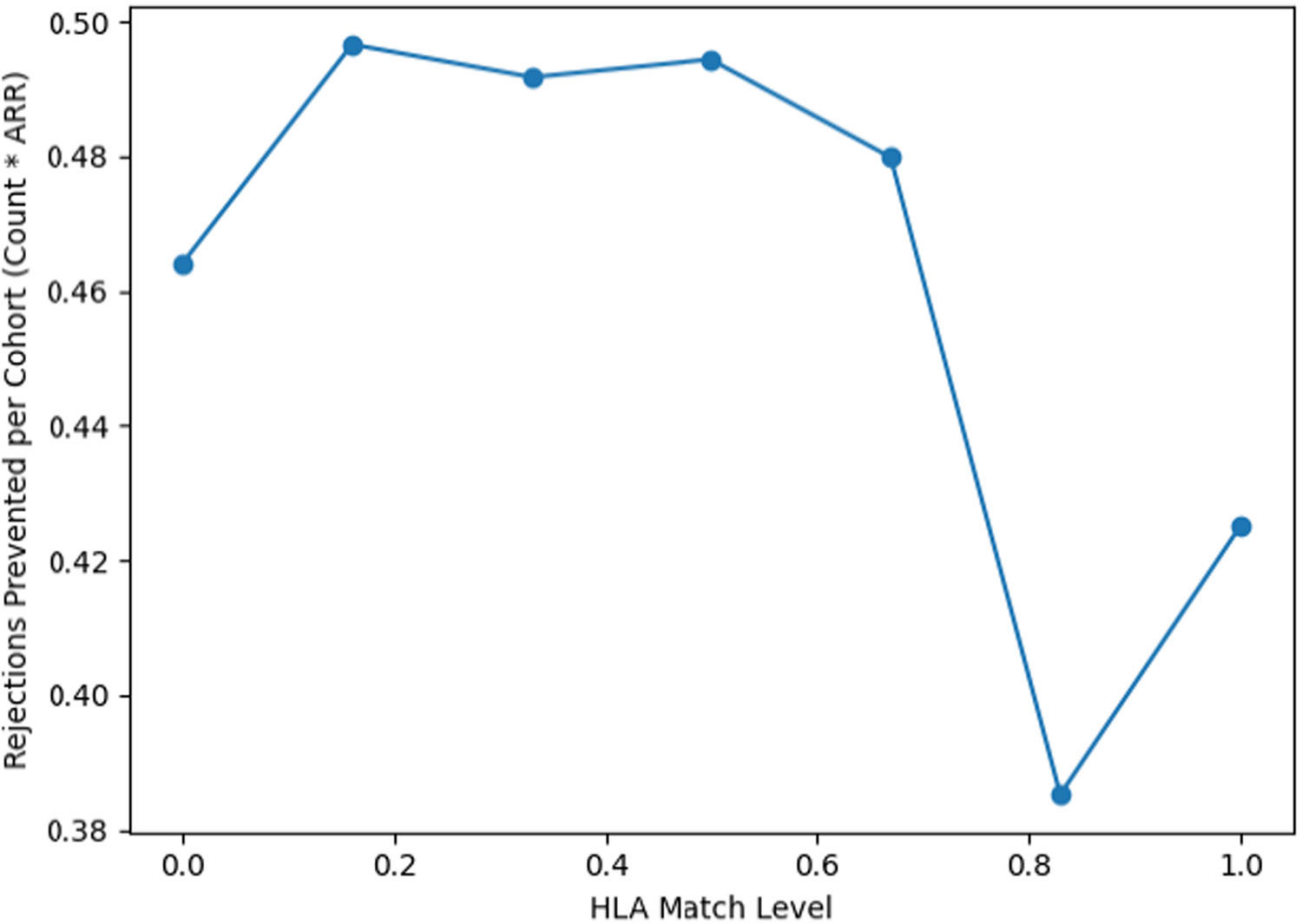
Cohort‐benefit of high dose by HLA match.

Figure 8 illustrates how the cohort‐benefit of high dose by match chart brings together both individual risk reduction and the size of each HLA‐match group to show how many rejection events are prevented in total by switching everyone in that group from the low‐dose to the high‐dose. While per‐patient ARR was highest at match group 0.83, that group has a sample of only 22 transplants. So, the total events prevented is 0.385. Conversely, the 0.16–0.50 match strata have both substantial ARR and large patient counts, yielding the greatest aggregate impact of nearly 0.5 rejections. The highest matches of 1 have few baseline events to prevent, and often smaller samples, so the total prevented cases drop again.

The differences in rejection benefit across HLA match levels are not only a reflection of biological compatibility but also of how many patients are in each match group. Figures 5 and 8 demonstrate this interaction. For example, some well‐matched groups show a relatively high‐risk reduction per patient, but because they include only a small number of transplants, the total number of prevented rejections remains low. On the other hand, groups with lower match levels often contain many more patients, so even if the individual risk reduction is smaller, the overall number of prevented rejection events is higher. In this way, variation in sample size helps explain why the apparent benefit of high‐dose therapy differs between match levels.

**Figure 9:**
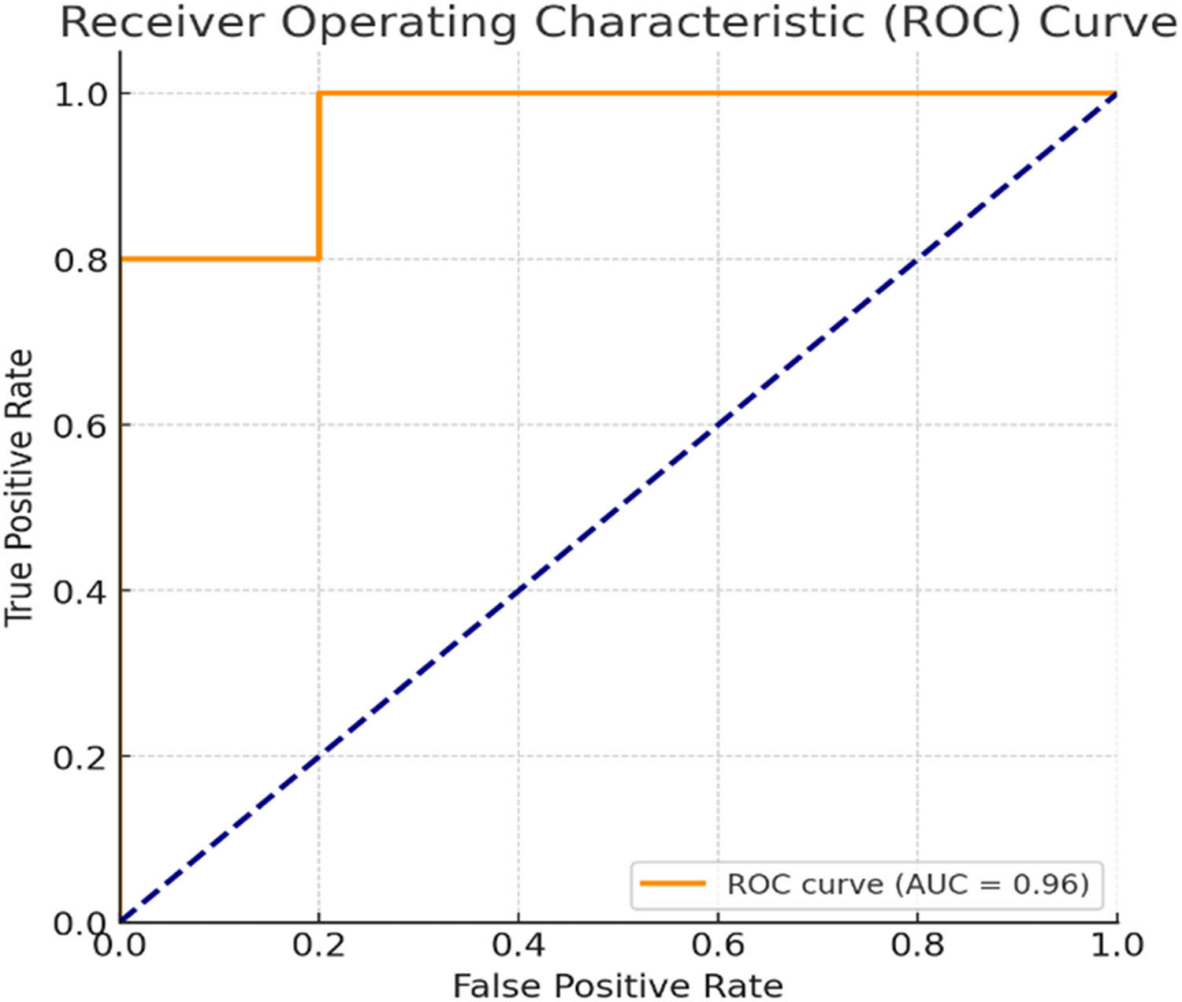
Receiver Operating Characteristic with Area Under the Curve.

Figure 9 demonstrates, the Receiver Operating Characteristic (ROC) analysis, that the constrained, regularized logistic regression models achieved excellent discrimination of rejection risk across HLA match levels, with an Area Under the Curve (AUC) of 0.96. This indicates that the fitted dose response functions reliably separate high‐ and low‐risk patients, consistent with the observed trends in ARR, RRR, and ERR. The steep rise of the curve confirms that sensitivity is maintained with minimal false positives, supporting the robustness of the modeling framework. Together with the ANOVA validation showing uniform dosing across match strata, these findings strengthen confidence that the observed variations in rejection risk reflect true immunologic effects of HLA compatibility rather than confounding from dose allocation.

In practical terms, this plot indicates that when the goal is to avert the greatest number of rejections overall with limited resources, the most impactful targets are those mid‐match recipients from 0.2 match to 0.6 match, where both the baseline risk and the group size align to maximize real‐world benefit.

## 4 Conclusion

This study shows that although all patients received the same baseline immunosuppressant dose regardless of their low‐resolution HLA match level, the additional benefit from higher dosing depends clearly on compatibility. Using a careful, multistage process, first checking data completeness and equal variances, then performing one‐way ANOVA and constrained ridge‐penalized logistic regression, it is confirmed that average dosing did not differ by match group, so dosing bias is ruled out. Crucially, the logistic models then quantify exactly how each increment in HLA match leads to both absolute and relative reductions in rejection risk: high‐dose treatment offers the largest extra benefit for poorly matched transplants, while well‐matched cases see only small gains, indicating diminishing returns.

These findings have two main implications. First, they endorse a risk‐based immunosuppression approach, where dosing can be adjusted not only for individual patient factors but also for match quality, potentially avoiding overtreatment in well‐matched recipients and directing higher doses where they achieve the greatest risk reduction. Second, they guide organ‐allocation policy by identifying match ranges (around 0.2–0.6) that combine sizable patient numbers with meaningful risk profiles to prevent the most rejections overall. This could inform the development of clinical decision support systems by providing quantitative risk estimates based on HLA match and initial tacrolimus dose. Similarly, organ allocation tools could incorporate these modeled risk reductions to prioritize donor–recipient pairs where small improvements in compatibility would yield the largest clinical benefit, helping optimize both patient outcomes and organ utilization.

This study is limited by a single‐center design and the use of low‐resolution HLA typing. Also, there is a lack of drug level monitoring and adherence data. Nepal only has a single hospital dedicated to organ transplantation where only low‐resolution HLA typing is available. The high‐resolution typing and eplept matching facilities are not yet established.

This study concentrated on low‐resolution HLA matching and tacrolimus dosing, but the same framework can be expanded to address current limitations. Incorporating additional immunologic markers such as DSA, PRA, eplet‐level mismatches, and non‐HLA antibodies and appropriate drug levels from robust monitoring could provide a more refined assessment of rejection risk, support more customized immunosuppressive strategies and potentially improve organ allocation. Achieving this, however, would require access to datasets with detailed immunologic profiles and standardized measures across patient groups. Further study also aims to define and validate rejection events more rigorously, incorporating both biopsy‐proven and clinically suspected cases which will enhance the accuracy and clinical relevance of outcome assessments in this analysis. This includes validation in larger, multicenter cohorts, which will also be important to ensure generalizability and strengthen individualized risk prediction. Finally, integrating these refined risk models into clinical decision support systems and organ allocation platforms could help move this study from analysis to real‐world practice.

## Author Contributions

**Samrajya Raj Acharya:** conceptualization, writing – original draft, methodology, investigation, visualization, formal analysis, software, validation, resources, data curation. **Aayush Man Regmi:** conceptualization, writing – original draft, methodology, investigation, visualization, formal analysis, software, validation, data curation. **Sushil Ghimire:** writing – review and editing, supervision. **Ram Prasad Ghimire:** supervision.

## Disclosure

The anonymized data required for this study were obtained in accordance with institutional protocols and upon payment of requisite data access fees from the only transplant centre in Nepal for research purposes independently. This study was conducted without any financial assistance.

## Ethics Statement

Granted by the Government of Nepal, Ministry of Health and Population, Shahid Dharma Bhakta National Transplant Centre (SDNTC) Research Review Committee, Reference Number: 35‐81/82.

## Consent

Consent is not available.

## Conflicts of Interest

The authors declare no conflicts of interest.

## Data Availability

The data sets generated and analyzed during the current investigation are available upon reasonable request from the author.
